# Abscisic Acid (ABA ) Promotes the Induction and Maintenance of Pear (*Pyrus pyrifolia* White Pear Group) Flower Bud Endodormancy

**DOI:** 10.3390/ijms19010310

**Published:** 2018-01-20

**Authors:** Jianzhao Li, Ying Xu, Qingfeng Niu, Lufang He, Yuanwen Teng, Songling Bai

**Affiliations:** 1 Department of Horticulture, Zhejiang University, Hangzhou 310058, China; 21316043@zju.edu.cn (J.L.); 21516057@zju.edu.cn (Y.X.); 11416008@zju.edu.cn (Q.N.); 2 The Key Laboratory of Horticultural Plant Growth, Development and Quality Improvement, the Ministry of Agriculture of China, Hangzhou 310058, China; 3 Zhejiang Provincial Key Laboratory of Integrative Biology of Horticultural Plants, Hangzhou 310058, China; 4 College of Life Sciences, Shaanxi Normal University, Xi‘an 710119, China; helufang@snnu.edu.cn (L.H.)

**Keywords:** ABA, bud dormancy, gene expression, metabolism, pear, signaling

## Abstract

Dormancy is an adaptive mechanism that allows temperate deciduous plants to survive unfavorable winter conditions. In the present work, we investigated the possible function of abscisic acid (ABA) on the endodormancy process in pear. The ABA content increased during pear flower bud endodormancy establishment and decreased towards endodormancy release. In total, 39 putative genes related to ABA metabolism and signal transductions were identified from pear genome. During the para- to endodormancy transition, *PpNCED-2* and *PpNCED-3* had high expression levels, while *PpCYP707A*s expression levels were low. However, during endodormancy, the expression of *PpCYP707A-3* sharply increased with increasing cold accumulation. At the same time, the ABA content of pear buds declined, and the percentage of bud breaks rapidly increased. On the other hand, the expression levels of *PpPYL*s, *PpPP2C*s, *PpSnRK2*s, and *PpABI4*/*ABI5*s were also changed during the pear flower bud dormancy cycle. Furthermore, exogenous ABA application to para-dormant buds significantly reduced the bud breaks and accelerated the transition to endodormancy. During the whole treatment time, the expression level of *PpPP2C-12* decreased to a greater extent in ABA-treated buds than in control. However, the expression levels of *PpSnRK2-1*, *PpSnRK2-4*, and *PpABI5-1* were higher in ABA-treated buds. Our results indicated that *PpCYP707A-3* and *PpNCED*s play pivotal roles on the regulation of endodormancy release, while ABA signal transduction pathway also appears to be involved in the process. The present work provided the basic information about the function of ABA-related genes during pear flower bud dormancy process.

## 1. Introduction

Bud dormancy is an adaptive mechanism of temperate deciduous fruit trees that allows them to survive detrimental winter environments [1]. During the dormancy stages, bud sprouting is repressed by external or internal signals. A well-accepted method classified the yearly dormancy cycle into three phases para-, endo-, and ecodormancy based on the repression signals [2]. The cessation of bud growth after shoot elongation stopped is known as paradormancy, which is controlled by the nearby organs/tissues, such as apical buds or adjacent leaves [2]. After leaves drop, the buds gradually switch to the endodormant state. In general, environmental cues, such as cool temperatures and short day-lengths, control endodormancy establishment [3]. In pears, low temperatures control bud growth cessation and dormancy induction [3]. The endodormancy state is acquired and maintained by the internal cues of the dormant buds. Therefore, even under optimal conditions, the buds cannot sprout. To complete endodormancy and resume growth, adequate chilling accumulation is required [4,5]. After the chilled hours fulfilled the chilling requirements, the pear trees gradually shift to the ecodormancy stage. In this stage, the buds rapidly sprout if they are in a favorable environment. Owing to global warming, many fruit trees, including pears, have suffered from abnormal weather conditions, including high temperatures in the autumn and warm winters, resulting in the interference of dormancy cycle-related transitions and affecting the production of the next growth season. Therefore, it is necessary to investigate the genetic factors underlying the control of dormancy to secure sustainable pear production [6].

Abscisic acid (ABA) is an essential plant hormone during the establishment, maintenance and release of seeds dormancy, as well as bud endodormancy. ABA-deficient mutants of *Arabidopsis thaliana* failed to develop seed dormancy [7]. Yamazaki et al. [8] found that an application of 500 µM ABA to *Allium wakegi* bulbs for 24 h significantly delayed their sprouting. When ABA increased in the growing shoot tips to a high of 900 µg/kg, shoot elongation ceased and terminal bud formation was induced [9]. The greatest concentration of free ABA in hazelnut buds was found in October (Spain) at the approximate beginning of endodormancy, followed by a decline in December, while it reached its lowest value before bud burst in March [10]. In grape bud, the concentration of ABA increased from para- to endodormancy, then declined during endodormancy release [11]. During the lateral flower bud dormancy in peach, the ABA level was high in endodormancy, reaching the lowest level in the period of ecodormancy [12]. Tuan et al. [13] found that in “Kosui”, pear (*Pyrus pyrifolia* Nakai), ABA concentration also increased during endodormancy then decreased during transition to ecodormancy.

The endogenous ABA content in plants is regulated by the balance between its biosynthesis and catabolism processes. During ABA biosynthesis, 9-*cis*-epoxycarotenoid dioxygenase (NCED) is the rate-limiting enzyme, which is primarily regulated at the transcriptional level [14,15]. During seed development and germination, *NCED6* and *NCED9* are responsible for ABA accumulation in *A. thaliana* embryos and endosperm. The over-expression of both genes results in a longer dormancy period [16]. The cytochrome P450 *CYP707A* family encodes the ABA 8-hydrolase, which catalyzes ABA by C-8 hydroxylation and produces 8-hydroxy ABA that is spontaneously isomerized in the phaseic acid [17]. In *Arabidopsis*, *CYP707A2* transcripts are highly expressed during the imbibition of seeds, which results in a decreased ABA level and dormancy release [18]. In potato (*Solanum tuberosum*), the expressions of *StNCED* and *StCYP707A* genes were found in correlation with changes in the ABA content in the meristem and cortex of the tubers. Furthermore, the decreased ABA content in the meristem is mostly related to tuber dormancy release [19]. Decreased ABA levels were recorded in leafy spurge during the transition from endo- to ecodormancy [20]. Decreased levels of ABA and ABA-biosynthetic genes expressions, and increased levels of ABA-catabolism genes were also found during the transition from endo- to ecodormancy in pear buds [21].

Dormancy is not only affected by ABA content but also by ABA-signaling pathway. Core ABA-signaling components consist of a group of ABA-receptor proteins, the REGULATORY COMPONENT OF ABA RECEPTOR (RCAR)/PYRABACTIN RESISTANCE(PYR)/PYRABACTIN RESISTANCE LIKE (PYL) family that act as negative regulators of the PROTEIN PHOSPHATASE 2C (PP2C, ABI1/ABI2) family. The interaction between PP2C and SNF1-related protein kinase 2 (SnRK2), which negatively affects ABA signaling, is disrupted by the binding of ABA to its RCAR/PYR/PYL receptors and the formation of ABA-receptor PP2C complexes. This allows the activation of SnRK2 and then activates downstream transcription factors (such as ABI4 and ABI5) that induce ABA-responsive genes expressions [22]. *ABI4* is a member of the APETALA2-domain gene family. The *Arabidopsis abi4* mutant shows an altered expression of ABA-responsive genes, such as *Em6* [23]. *ABI5* is a basic leucine zipper (bZIP) transcription factor that is involved in regulating germination in response to ABA and stress [24].

Although the effect of ABA on pear bud dormancy has been observed [13], the detailed regulatory pathway remains obscure. In this work, we investigated the ABA content and expression patterns of ABA-related genes during pear flower bud dormancy. Furthermore, we determined the roles of ABA on promoting endodormancy induction by applying exogenous ABA to para-dormant pear flower buds. Our results indicated that *PpCYP707A-3* might be an important regulator of endodormancy release, and some other ABA-related genes also contributed to this biological process.

## 2. Results

### 2.1. Daily Temperatures, Bud-Break Percentages, and ABA Content during the Natural Flower Bud Dormancy Cycle

**Figure 1:**
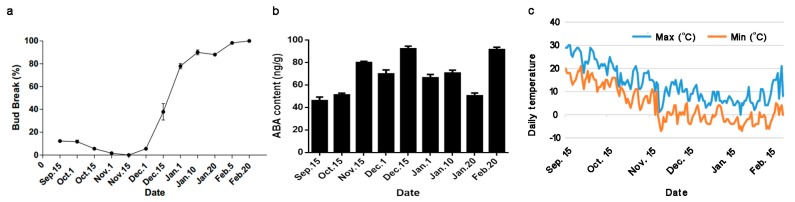
The change of abscisic acid (ABA) content during the endodormancy period. (**a**) Dormant shoots of field-grown “Suli” pear trees were collected from 15 September 2016 to 20 February 2017, then kept in water in a phytotron at day/night temperatures of 25 ± 1/22 ± 1 °C, with a 12-h photoperiod of white light (320 μmol photon m^−2^·s^−1^), and 75% relative humidity. Percentage of bud breaks was assessed after 21 days using 12 shoots per sampling period. Each bar represents the mean ± SEM (*n* = 3). (**b**) The concentration of ABA in pear flower buds at different dormant states. Each bar represents the mean ± SEM (*n* = 3). (**c**) Daily maximum and minimum temperatures during 2016–2017.

In Dangshan County, the leaves of “Suli” pear usually fall in late October, and the trees shift to endodormancy. The dormancy states of “Suli” flower buds during the endodormancy had been determined in 2011–2012 [25]. In that study, the pear trees had already transitioned to endodormancy status (bud break under forcing conditions = 0%) before 15 November. After chilling accumulation, the endodormancy was released in early January when the bud-break rate (under forcing conditions) was over 50%. To further confirm the relationship between ABA and pear bud dormancy, we monitored the dormancy status and ABA content changes in 2016–2017. As shown in the Figure 1a, the deep endodormancy in this season started from early November and was released in middle-late December. The ABA content increased rapidly from para- to endodormancy, and then declined during the release of endodormancy (Figure 1b), suggesting that the depth of endodormancy is correlated to the ABA content. To discriminate whether the changes of ABA content was the response to the low temperature, we also checked the daily temperature during dormancy (Figure 1c). The maximum and minimum temperatures declined from 15 September to 15 November, and then the daily temperature remained roughly constant in a low level to the early February. The trends in temperature and ABA content were significantly different, especially after 15 November when the temperature did not significantly change but the ABA content increased to the peak at 15 December then decreased rapidly. Thus, the change in the ABA content was not just simply a response to low temperatures. Interestingly, we observed that ABA content increased at 20 February with a suddenly temperature decrease (Figure 1b,c).

### 2.2. Identification of ABA Metabolism- and Signaling-Related Genes in Pear

**Figure 2:**
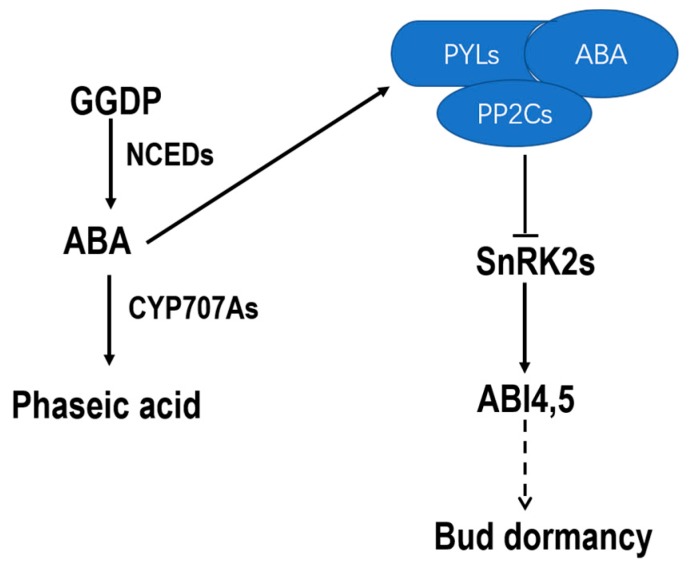
Model of ABA biosynthesis, metabolism, and signaling in pear modified according to *Arabidopsis thaliana*. ABA is synthesized from geranylgeranyl diphosphate (GGDP) by 9-*cis*-epoxycarotenoid dioxygenases (NCEDs), and then degraded by CYP707As to phaseic acid. ABA is accepted by PYLs, and ABA–PYL complexes can inhibit PP2C functions. PP2Cs can suppress SnRK2s function through dephosphorylating. Phosphorylated SnRK2s can active ABI4 and ABI5. ABI4 and ABI5 accumulate to regulate the downstream genes expression when the plant accumulates higher level ABA.

To further identify the role of ABA in the regulation of pear flower bud dormancy, we identified the genes involved in ABA metabolism and signal transduction (Figure 2). We retrieved the protein sequences of NCEDs of *Vitis vinifera*, CYP707As, ABI1, ABI2, ABI4, ABI5, and PYLs of *A. thaliana*, and SnRK2s of *Malus domestica* from NCBI to use as query for a local BLAST algorithm-based search against the pear genome. Finally, 14 *PP2C* genes, 1 *ABI4* gene, 2 *ABI5* genes, 3 *NCED* genes, 6 *SnRK2* genes, 5 *CYP707A* genes and 8 *PYL* genes that potentially involved in ABA metabolism and signaling were selected. These genes were named based on their homologues and numbered according to chromosomal location when multiple genes were identified in a same gene family (Table 1).

### 2.3. Expression Patterns of ABA Synthesis, Catabolism, and Signaling Genes during Bud Dormancy

**Figure 3:**
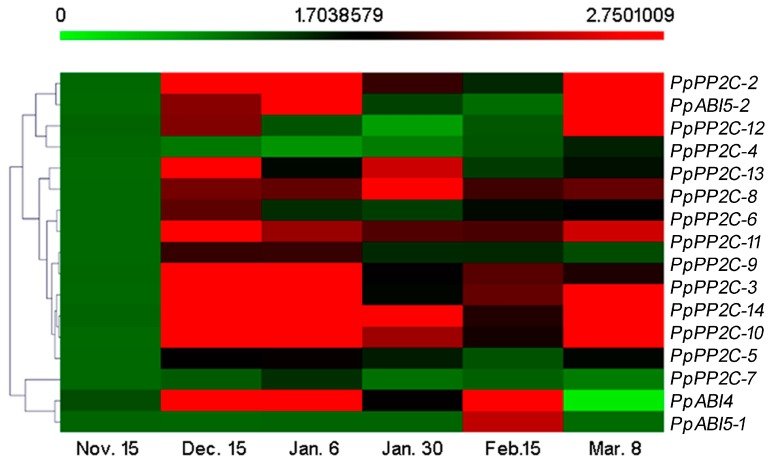
Relative gene expression levels of 17 *PP2C*s and *ABI4*/*5*s genes during flower bud dormancy in 2011–2012. Expression values were normalized to the value of the *PpACTIN* gene. Each value represents the average of three biological replicates. The sampling dates were 15 November, 15 December, 6 January, 30 January, 15 February, and 8 March.

To identify the genes that may be involved in regulating dormancy transition, the transcript profiles of *PP2C*s, *ABI4*, and *ABI5*s genes were first investigated using the 2011–2012 floral buds of “Suli” pear during dormancy (Figure 3). Among these 17 genes, most of the *PP2C* genes’ expression levels increased during the endodormancy, and then decreased during ecodormancy, but increased before bud break. Near the endodormancy release period, the expression levels of *PpPP2C-1*/*2*/*5*/*7*/*9*/*10*/*14* reached their peaks. *PpPP2C-3*/*6*/*11*/*12*/*13* peaked on 15 December. The expression patterns of the transcription factors *PpABI4* and *PpABI5-2* were the same as those of *PpPP2C-1*/*2*/*5*/*7*/*9*/*10*/*14*, peaking during the endodormancy transition to ecodormancy. However, the expression of *PpABI5-1* did not obviously change, but had a peak on 15 February.

**Figure 4:**
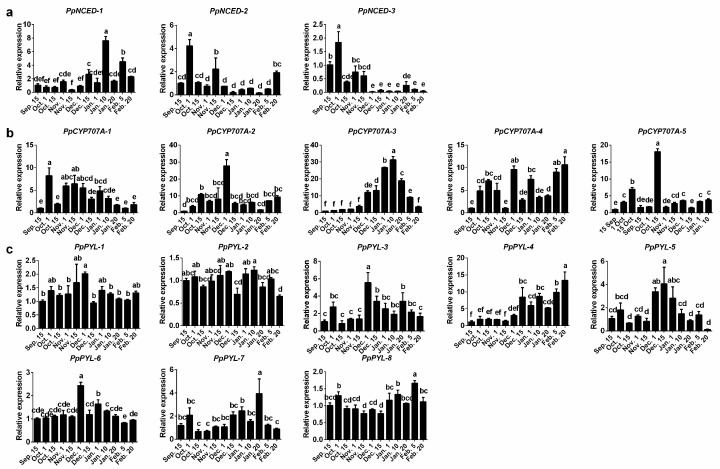
Relative gene expression levels of ABA metabolism and receptor genes during pear flower bud dormancy on 2016–2017. Expression values were normalized to the value of the *PpACTIN* gene. Each bar represents the mean ± SEM of three biological replicates. Different letters indicate a significant difference among different dates (*p* < 0.05).

**Figure 5:**
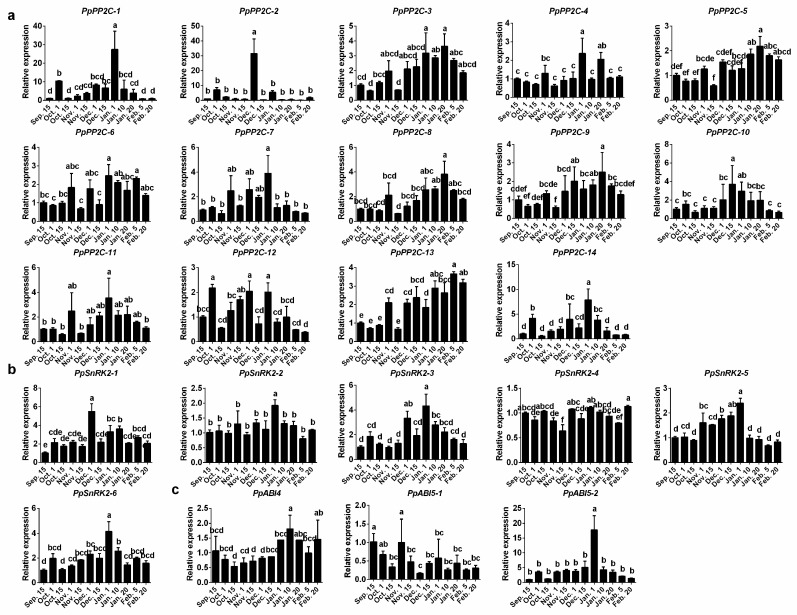
Relative gene expression levels of ABA signaling transduction genes ((**a**) PP2Cs; (**b**) SnRK2s; (**c**) ABI4/ABI5) during flower bud dormancy on 2016–2017. Expression values were normalized to the value of the *PpACTIN* gene. Each bar represents the mean ± SEM of three biological replicates. Different letters indicate a significant difference among different dates (*p* < 0.05).

Furthermore, we determined the expression patterns of ABA metabolism and signaling genes using the buds collected in the 2016–2017 season. Among these genes, the expression of *PpCYP707A-3* rapidly increased during chilling accumulation and fast declined toward endodormancy release (Figure 4b). The expression levels of *PpNCED-2* and *PpNCED-3* were higher at the paradormancy, and then dramatically decreased to lower levels during endodormancy and ecodormancy period (Figure 4a). On the contrary, *PpNCED-1* slowly increased during endodormancy, peaked on 10 January, and decreased thereafter (Figure 4a). The expression levels of *PpPYL-1*/*3*/*5* genes were consistent with the ABA content (Figure 4c). In addition, we found that the expression levels of *PpPP2C-1*/*3*/*5*/*8*/*9*/*10*/*11* were downregulated during endodormancy and then upregulated during endodormancy release, similar with *PpCYP707A-3* (Figure 5a). The expression level changes of *SnRK2-3*/*5*/*6* and *PpABI5-2* were similar as those of *PpPYL-1*/*3*/*5* (Figure 5b,c).

### 2.4. Effects of Exogenous ABA on the Endodormancy-Related Induction of Pear Flower Buds

**Figure 6:**
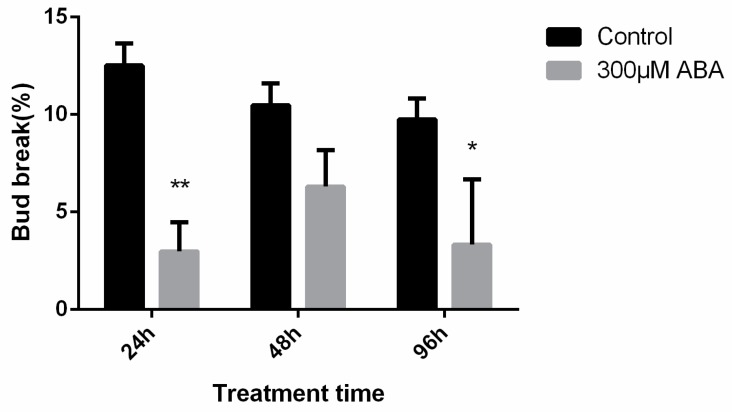
ABA promotes the induction of pear lateral flower bud endodormancy. The pear shoots were collected on 15 October 2016. The shoots were dipped in 300 µM ABA or control (water with 0.02% Triton X-100) for 24, 48, and 96 h. After the ABA treatment, shoots were placed in water for the next 21 days under forcing conditions before measuring the bud-break percentage. The bud-break experiments were performed with three biological replicates and each bar represents the mean ± SEM. Different number of asterisks indicate a significant difference among different treatment time according to Student’s *t*-test (* *p* < 0.05, ** *p* < 0.01).

To further analyze the function of ABA on the induction of endodormancy, exogenous different concentrations ABA (100, 200, and 300 μM) were applied to paradormant pear flower buds collected on 15 October 2016, and the responses of dormant buds to applications of exogenous ABA or water were compared (Figure 6 and Figure S1). ABA applications had a significantly promotive effect on the endodormancy induction of the pear buds. The incubation of pear flower bud shoots with 100, 200, and 300 μM ABA for 24, 48, and 96 h resulted in decreases of ~5% in bud break percentages relative to the control. The bud-break percentages of different ABA concentrations and treatment times were similar; therefore, we only chose to use 96-h ABA-treated materials in further experiments.

### 2.5. Effects of Exogenous ABA on Pear ABA Metabolism- and Signaling-Related Gene Expression Levels

**Figure 7:**
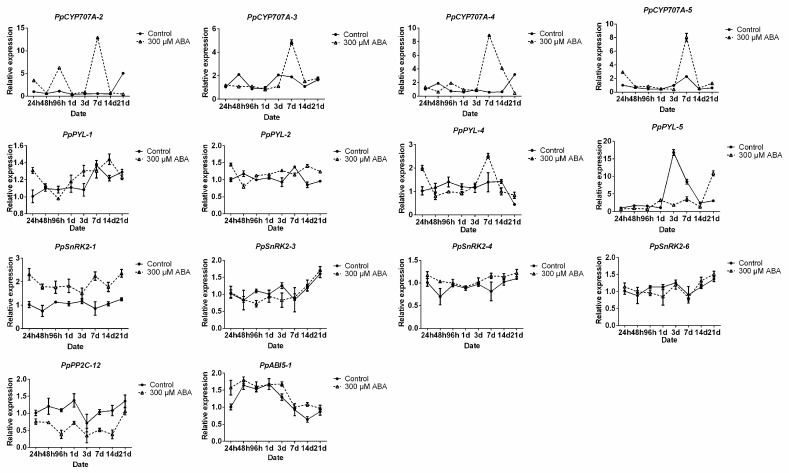
Exogenous ABA modulate the transcription of ABA-responsive genes. Total RNA was extracted from the control and 300-µM ABA-treated buds sampled at 24, 48, and 96 h after beginning of ABA-treatment, as well as 1, 3, 7, 14, and 21 days after treatment. Relative expression levels of ABA-responsive genes were determined by qRT-PCR as described in the Materials and Methods, and normalized against *PpACTIN*. The bars represent the mean ± SEM of three biological repeats.

The lower bud-break rate of ABA-treated shoots indicated that ABA accelerated the induction of dormancy in lateral flower buds. The expression levels of ABA-related genes were further tested. The expression of *PpNCEDs* were not significantly altered after ABA treatment (Figure S2). However, the transcription abundances of *PpCYP707A-2*, *PpCYP707A-3*, *PpCYP707A-4*, and *PpCYP707A-5* dramatically increased in ABA-treated buds on the 7th day after treatment (Figure 7). *PpPYL-1*, *PpPYL-2*, *PpPYL-4*, and *PpPYL-6* expression levels in ABA-treated buds increased just 24 h after treatment compared with that in the control, and then the expression levels were similar. *PpPP2C-12* expression level decreased to a greater extent in ABA-treated buds than in water-treated buds. During the whole treatment time, the expression levels of *PpSnRK2-1*, *PpSnRK2-4*, and *PpABI5-1* were higher in the treatment than in control. The mRNA levels of *PpSnRK2-3* and *PpSnRK2-6* were upregulated during the forcing; however, no significant differences in expressions between control and treatment were observed after changing to water.

## 3. Discussion

ABA is involved in maintaining bud dormancy in woody plants; however, there is limited molecular data supporting this observation [26,27,28]. A gradual decrease in the ABA content during the transition from endo- to ecodormancy has been reported previously in leafy spurge and pear buds [13,29]. Treatment with exogenous ABA induces bud dormancy and prevents its release in apple and grape [11,30]. It also induces dormancy in the epiphyllous buds of *Kalanchoe tubiflora* [31]. In isolated rose buds treated with fluridone, an inhibitor of carotenoid synthesis, a significant decrease in the endogenous ABA content occurred, preventing dormancy onset [32]. Exogenous ABA applications reversibly inhibit the sprouting of Taiwanese pear (*Pyrus pyrifolia* “Hengshanli”) leaf buds in the ecodormancy stage [21]. Here, the ABA content in the “Suli” pear lateral flower buds also increased from para- to endodormancy, reaching to a maximum before the bud-break percentage started to increase and then decreased along with the endodormancy release (Figure 1b). Thus, a close relationship was observed between the ABA content and pear flower bud dormancy. On the other hand, the ABA treatment inhibited the break of para-dormant bud, which agreed with the observation of the increase of ABA content during endodormancy establishment (Figure 1b and Figure 6), suggesting that ABA may play an important role in the induction of pear flower bud deep dormancy (Figure 6 and Figure S1). However, the inhibition was dosage independent, which is different from that in grape, where high concentration ABA inhibited bud breaks more efficiently [11]. In this work we provided the information supporting the hypothesis that ABA is a positive regulator of endodormancy induction and a negative regulator of endodormancy release. Interestingly, we found that the ABA content had a peak before budburst. This may be a protective strategy for pear to survive under suddenly decreased temperature on those days as shown in Figure 1c, but the mechanism needs to be further studied.

Although ABA is an important phytohormone for bud dormancy, the functions of ABA biosynthesis, catabolism, and signal transduction genes on bud dormancy regulation have not been described in detail. Transcriptomic study on Japanese pear supported the important roles of the *CYP707A*s and *NCED*s [21]. In *Arabidopsis*, *AtNCED6* and *AtNCED9* are major players in the regulation of seed dormancy, and the double-mutant undergoes reduced seed dormancy [16]. In barley, transcript levels of *HvNCED2*, but not *HvNCED1*, increased during grain development from early to middle stages and modulate ABA accumulation [33]. Although seed dormancy and bud dormancy are different biological processes, the molecular mechanisms are similar in some aspects [12]. Ruttink et al. [34] found that *NCED3* was upregulated under short-day-induced endodormancy conditions just prior to endodormancy establishment in poplar. In our results, *PpNCED-2* and *PpNCED-3* expression levels were higher at the entrance to endodormancy and then dramatically decreased to lower levels during the endo- to ecodormancy period (Figure 4a). However, *PpNCED-1* increased slowly during endodormancy, peaked at 10 January, and then decreased during transition to ecodormancy. Similarly, in grape, the ABA content in buds was also consistent with the *NCED* expression pattern, peaking during endodormancy, and gradually decreasing during the transition to ecodormancy [11].

During the decline of daily temperature before 15 November, the expression of *PpCYP707A-3* did not significantly change. However, during chilling accumulation, when the temperature did not have an evident variation, the expression of *PpCYP707A-3* greatly changed. Specifically, its expression increase was parallel with cold accumulation. At the same time, the ABA content of pear bud declined. On the contrary, the percentage of bud break increased rapidly. According to these results, we infer that *PpCYP707A-3* may be related to endodormancy release. We further compared the results with our transcriptomic data [35], and found that the similar expression pattern of *PpCYP707A3* in the winter 2011-2012. Similarly, *PpCYP707A* had a high expression level during the transition from endo- to ecodormancy in the Japanese pear “Kosui” [13]. These results were similar with those of peach, in which *PpeCYP707A3* was highly expressed during the endodormancy release, leading to a lower ABA content [12]. We compared the protein sequences of three peach CYP707As with *PpCYP707A-3*. The results showed that *PpCYP707A-3* had 68% similarity with *PpeCYP707A3*, other two shared only 50% similarity (Figure S3), suggesting that *PpCYP707A-3* and *PpeCYP707A3* may have similar functions. On the other hand, CYP707A also regulate dormancy progress on other organs/tissues. In barley, *HvCYP707A1* responded to changes in environmental conditions decreasing the ABA level at the late maturation stages [33]. In potato tuber, decreased in the ABA content correlated mainly with *StCYP707A2* [36]. Therefore, we propose that *PpCYP707A-3* plays an important role in regulating endodormancy release.

ABA signal transduction is a key process in the regulation of seed dormancy. The ABA receptors PYR/PYL/RCAR negatively regulate ABA signaling by inhibiting the PP2C protein phosphatases [37,38]. Kim et al. [39] found that the overexpression of *OsPYL*/*RCAR5* in rice led to later seed germination, suggesting a positive role in seed dormancy regulation. The transcriptomic analysis of bud dormancy in grape showed that transcripts related to the PP2C family are upregulated in paradormancy as compared to endodormancy [40]. Poplar overexpressing the *Arabidopsis* ABA insensitive 1 (*ABI1*) gene resulted in the insensitivity to exogenous ABA applications during the outgrowth of buds [41]. In this work, we observed the increased expression of *PpPYL-1*/*3*/*5* during the transition from para- to endodormancy (Figure 4c). Additionally, the expression levels of *PpPP2C-3*/*5*/*8*/*9*/*13*, which negatively regulate ABA-signaling pathway, increased in parallel with decreases in the ABA content (Figure 5a). These results suggest that these genes may take part in dormancy regulation.

In plants, PP2Cs (including *ABI1*/*ABI2*) negatively regulate the ABA-signaling pathway through a dephosphorylated SnRK2 subclass III kinase [42], while the phosphorylated *SnRK2*s can activate downstream bZIP transcription factors (AREB/ABFs), such as *ABI4* and *ABI5* that are positive regulators of ABA responses during seed germination [43], to activate ABA signaling [44]. Fujii et al. [45] and Nakashima et al. [46] found that *SnRK2.2*/*SnRK2.3*/*SnRK2.6* double or triple mutants all decreased seed dormancy in *Arabidopsis*. In our experiments, the expression patterns of *SnRK2*s were different in naturally dormant and ABA-treated buds. During natural bud dormancy, *SnRK2-3*/*5*/*6* increased from para- to endodormancy, but the higher expressed genes in ABA-treated buds were *PpSnRK2-1* and *PpSnRK2-4* (Figure 5 and Figure 7). Furthermore, *PpABI4* and *PpABI5-2* expression levels significantly increased from para- to endodormancy in pear flower buds (Figure 3 and Figure 5c), while the *PpABI5-1* gene did not significantly change. Overall, some ABA signal transduction related genes showed the expression change during the endodormancy process, suggesting that they were under transcriptional regulation during endodormancy. However, we cannot exclude the involvement of other genes in the regulation of endodormancy process, as most of these genes subjected to the regulation in posttranslational level, their detailed functions need to be further studied.

## 4. Materials and Methods

### 4.1. Plant Materials

Thirty-year-old “Suli” pear trees (*Pyrus pyrifolia* white pear group) grafted onto *Pyrus betulaefolia* Bunge rootstocks in the Dangshan Germplasm Resources Center (Dangshan County, Suzhou, Anhui Province, China) were used in this study. These trees were not pruned or chemically treated prior to treatment. All of the bud samples were collected from the same trees at each dormancy stage, immediately frozen in liquid nitrogen, and stored at −80 °C until use. Transcript and expression analyses were performed on lateral flower buds collected during two different dormancy spans, from November 2011 to March 2012, at 15 November, 15 December, 6 January, 30 January, 15 February, and 8 March, and from September 2016 to February 2017 at 15 September, 1 October, 15 October, 1 November, 15 November, 1 December, 15 December, 1 January, 10 January, 20 January, 5 February, and 20 February. The buds collected from nine trees were used as three biological replicates.

### 4.2. Measurement of Lateral Flower Bud’ Dormancy Status

The dormancy status of lateral flower buds was estimated as described previously [35]. Twelve one-year-old shoots, approximately 60-cm long and bearing apical buds and 10–12 lateral flower buds, were collected and placed in water in 500-mL vials in a phytotron and kept under a day/night temperature of 25 ± 1/22 ± 1 °C, with a 12-h photoperiod of white light (320 µmol photon m^−2^·s^−1^) and 75% relative humidity. The water in the vials was changed, and the basal ends of the shoots were cut every 3–4 days. After 21 days, the dormancy status was evaluated by determining the percentage of bud breaks. The beginning of bud break was defined as green leaf tips enclosing visible flowers [47] (Figure S4). Lateral flower buds, after 21 days cultivated in water, with more than 50% bud breaks, were considered as endodormant release [48].

### 4.3. Daily Temperature during Winter

The environmental maximum and minimum temperatures during the entire winter in the city (Dangshan County) were determined from http://lishi.tianqi.com/dangshan/index.html.

### 4.4. ABA Treatments

The shoots of “Suli” pear, collected on 15 October 2016 from the Dangshan Germplasm Resources Center, were dipped in 300 µM ABA (RYON, Shanghai, China) solution supplemented with 0.02% (*v*/*v*) Triton X-100 for 4 days. After ABA treatments, the shoots were placed in water in a controlled phytotron as mentioned above for 21 days to record the rate of bud break. Buds were collected 24, 48, and 96 h after beginning of ABA treatment, and 1, 3, 7, 14, and 21 days after moved to water. The collected buds were frozen in liquid nitrogen, and stored at −80 °C until used. In addition, shoots soaked in water with 0.02% Triton X-100 solution were used as control.

### 4.5. Identification of Genes Related to ABA Biosynthesis, Metabolism, and Signaling

The protein sequences of NCEDs (NP_001268199.1, NP_001268200.1, AFP28804.1) of *Vitis vinifera*, CYP707As (NP_567581.1, NP_180473.1, NP_851136.1, NP_566628.1), ABI1 (NP_194338.1), ABI2 (NP_200515.1), ABI4 (NP_181551.1), ABI5 (NP_565840.1), and PYLs (NP_199491.2, NP 180174.1, NP 177443.1, NP565887.1, NP 565928.1, NP 194521.2, NP199398.1, NP199399.1, NP 193597.1) of *A. thaliana*, and SnRK2s (AII82266.1, AII82268.1, AGC31662.1, AII82269.1, AII82270.1, AII82271.1, AII82272.1, AII82273.1) of *Malus domestica* were downloaded from NCBI (https://www.ncbi.nlm.nih.gov/) and used as query to perform the local BLAST (version 2.2.20, NCBI Bethesda, MD, USA.) against pear genome [49]. The protein sequences of the candidate homologues (*e* < 1 × 10^−10^) of each gene were retrieved from the pear genome database. Then, we ran a multiple sequence alignment in DNAMAN (version 7, Lynnon Corporation, Quebec, QC, Canada) to identify the similarities among each gene cluster. If the identities of two proteins were more than 95%, then we selected only one for further experiments.

### 4.6. Determination of ABA Concentration

The extraction and purification of ABA, as well as the determination of its content were performed as described by Niu et al. [50] with slight modifications. Crude plant extracts were prepared using ethyl acetate containing an internal standard (10 ng of (^2^H_6_) ABA, Toronto Research Chemicals, Toronto, ON, Canada), then purified with a Sep-Pak™ C18 reversed-phase extraction cartridge. Approximately 0.3 g samples per extraction were used to determine the ABA content using ultra-performance liquid chromatography/mass spectrometry/mass spectrometry (UPLC/MS/MS).

### 4.7. RNA Isolation and Quantitative Real-Time RT-PCR Analysis

Total RNA was extracted using the modified Cetyltrimethyl Ammonium Bromide (CTAB) method [51]. The RNA solutions were subjected to an extra chloroform extraction and ethanol precipitation at −20 °C overnight. First-strand cDNA was synthesized from 1 µg RNA using the PrimeScript™ RT reagent Kit with gDNA Eraser (Perfect Real Time; Takara Biotechnology (Dalian) Co., Ltd. Dalian, China) according to the manufacturer’s instructions. The cDNA was used as the template for qRT-PCR. The primer sequences (designed using Primer3, http://bioinfo.ut.ee/primer3-0.4.0/) are listed in Table S1. Primer efficiencies were tested using a standard curve for each gene, and only primers with efficiency levels between 80% and 120% were used for qRT-PCR. The measurements were obtained using the relative quantification method (2^−ΔΔ*C*t^), and the gene transcript levels were normalized to that of the *actin* gene (PpActin, JN684184; [35]).

### 4.8. Statistical Analysis

The experiment was organized using a completely randomized design. The data were subjected to ANOVA followed by least-significant difference tests. Statistically significant differences were indicated at *p* < 0.05 or *p* < 0.01 level. All data were analyzed using the statistical package for social sciences software (SPSS v.18.0, IBM, Armonk, NY, USA).

## 5. Conclusions

In conclusion, ABA content in pear buds was correlated to the endodormancy process; the expression of *PpCYP707A-3* might highly respond to chilling accumulation, suggesting its pivotal role on the regulation of endodormancy release. The expression levels of *PpNCED*s, *PpPYL*s, *PpPP2C*s, *PpSnRK2*s, and *PpABI4*/*ABI5*s also significantly changed during the pear flower bud dormancy cycle. These results indicate that ABA is involved in regulating bud dormancy through its metabolism and signal-transduction pathways. However, posttranslational level of the main ABA signal transduction components, such as PP2Cs and SnRK2s, were not investigated in the present work, their detailed functional mechanisms need to be further elucidated.

## Figures and Tables

**Table 1:** Abscisic acid (ABA) biosynthesis, metabolism, and signaling genes located in the pear genome.

Gene Name	Genome Locus Tag	Chromosome Location	Start	End
*PpPP2C-1*	Pbr013576.1	Chr1	9763088	9766905
*PpPP2C-2*	Pbr041497.1	Chr2	18358353	18360378
*PpPP2C-3*	Pbr013336.1	Chr3	20846735	20850224
*PpPP2C-4*	Pbr028942.1	Chr4	1769534	1772968
*PpPP2C-5*	Pbr009703.1	Chr7	1815301	1820662
*PpPP2C-6*	Pbr025010.1	Chr8	14605450	14607580
*PpPP2C-7*	Pbr015257.1	Chr9	7829375	7832873
*PpPP2C-8*	Pbr041795.2	Chr12	14167470	14172092
*PpPP2C-9*	Pbr028792.1	Chr13	1840077	1843505
*PpPP2C-10*	Pbr026157.1	Chr14	6552129	6556046
*PpPP2C-11*	Pbr019878.1	Chr15	6270769	6272939
*PpPP2C-12*	Pbr015521.1	Chr15	15615471	15619039
*PpPP2C-13*	Pbr019599.1	Chr15	8273643	8277518
*PpPP2C-14*	Pbr022745.1	Scaffold346.0.1	139061	141715
*PpABI4*	Pbr009544.1	Chr1	7155273	7156489
*PpABI5-1*	Pbr017778.1	Chr12	20360616	20363301
*PpABI5-2*	Pbr007589.1	Chr14	344099	346885
*PpNCED-1*	Pbr039596.1	Chr10	6419191	6421260
*PpNCED-2*	Pbr009089.1	Chr10	10254167	10256724
*PpNCED-3*	Pbr006012.1	Chr16	10335999	10337804
*PpSnRK2-1*	Pbr040276.1	Chr6	16755401	16758728
*PpSnRK2-2*	Pbr026536.1	Chr8	4160916	4164639
*PpSnRK2-3*	Pbr040625.1	Chr15	36546195	36549943
*PpSnRK2-4*	Pbr003186.1	Chr15	41233666	41237650
*PpSnRK2-5*	Pbr023607.1	Scaffold364.0	289329	293241
*PpSnRK2-6*	Pbr042784.1	Scaffold364.0	337528	342127
*PpCYP707A-1*	Pbr003860.1	Chr3	26178912	26186900
*PpCYP707A-2*	Pbr006776.1	Chr6	18879003	18881665
*PpCYP707A-3*	Pbr019636.1	Chr15	7961660	7964906
*PpCYP707A-4*	Pbr029414.1	Chr16	18720527	18723493
*PpCYP707A-5*	Pbr004630.1	Scaffold1214.0	40622	43660
*PpPYL-1*	Pbr027457.1	Chr1	3660265	3662621
*PpPYL-2*	Pbr013616.1	Chr1	9469866	9472306
*PpPYL-3*	Pbr000497.1	Chr5	25031100	25031660
*PpPYL-4*	Pbr036422.1	Chr8	16927910	16928515
*PpPYL-5*	Pbr016128.1	Chr10	3588258	3588818
*PpPYL-6*	Pbr010794.1	Chr13	185820	188146
*PpPYL-7*	Pbr019827.1	Chr15	6628678	6629283
*PpPYL-8*	Pbr042468.1	Scaffold984.0	68401	69066

## Supplementary Materials

Supplementary materials can be found at www.mdpi.com/1422-0067/19/1/310/s1.

## Abbreviations

ABA	Abscisic acid
NCED	9-*cis*-Epoxycarotenoid dioxygenase
CYP707A	ABA 8′-hydroxylase
RCAR/PYR/PYL	Regulatory component of aba receptor/pyrabactin resistance/pyrabactin resistance like
PP2C	Protein phosphatase 2C
SnRK2	Snf1-Related kinase 2
ABI	Abscisic acid insensitive
GGDP	Geranylgeranyl diphosphate
CTAB	Cetyltrimethyl Ammonium Bromide
