# Supplementary material for: Abscisic Acid (ABA ) Promotes the Induction and Maintenance of Pear (Pyrus pyrifolia White Pear Group) Flower Bud Endodormancy
Source: Int J Mol Sci. 2018 Jan 20;19(1):310. doi: 10.3390/ijms19010310 (PMC5796254; doi:10.3390/ijms19010310)
Supplement: Supplementary file 1 [file ijms-19-00310-s001.pdf]

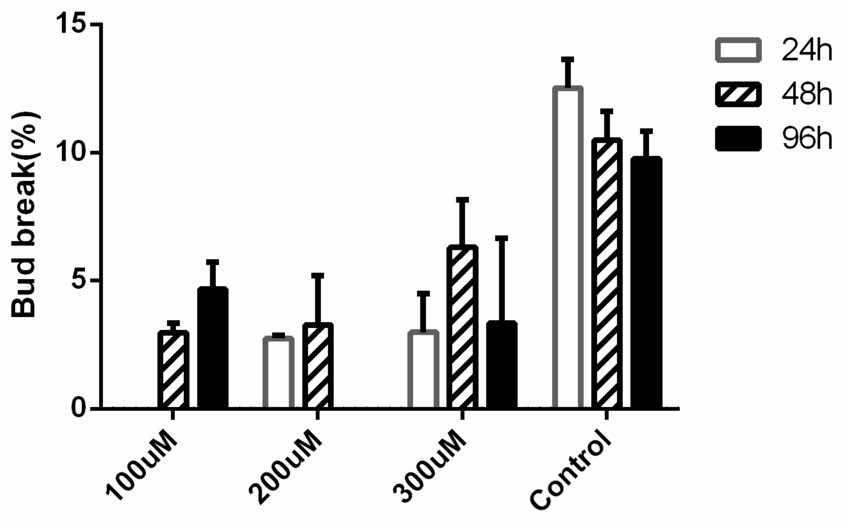

Figure S1. ABA promotes the induction of pear lateral flower bud endo-dormancy. The shoots were collected on 15 October, 2016. The shoots were dipped in 100, 200 and 300 μM ABA or water with 0.02% Triton X-100 (control) for 24, 48, and 96 h. After the ABA treatment, shoots were placed in water for the next 21 days under forcing conditions before measuring the bud-break percentage. The bud-break experiment was performed with three biological replicates and each bar represents the mean ± SEM.

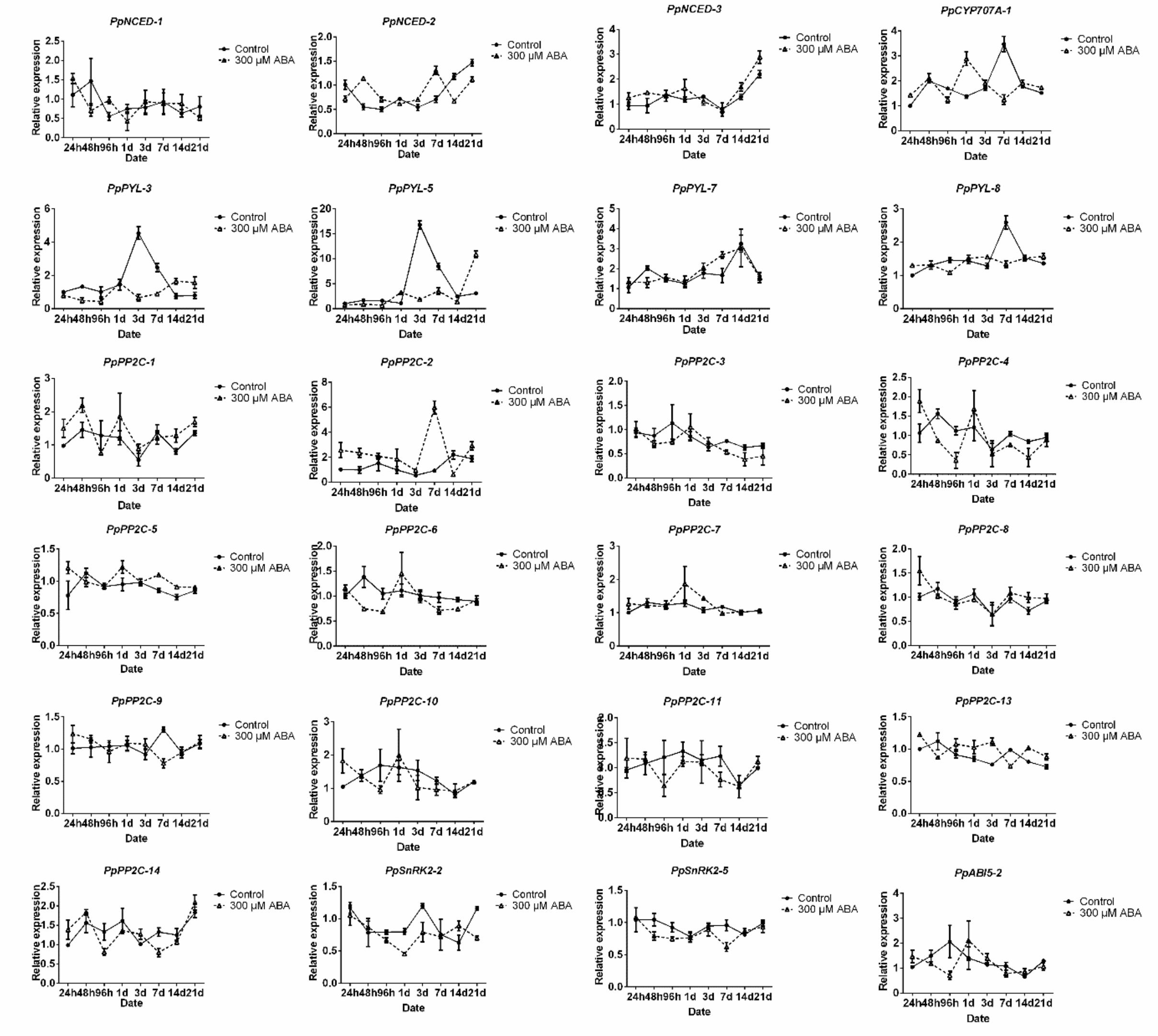

Figure S2. Dormancy-induced stimuli modulate the transcription of ABA-responsive genes. Total RNA was extracted from the control and 300 μM ABA-treated buds sampled at 24, 48, and 96 h, as well as 1, 3, 7, 14, and 21days after treatment. Relative expression levels of ABA-responsive genes were determined by qRT-PCR as described in the Materials and Methods, and normalized against *PpACTIN*. The bars represent the mean ± SEM of three biological repeats.

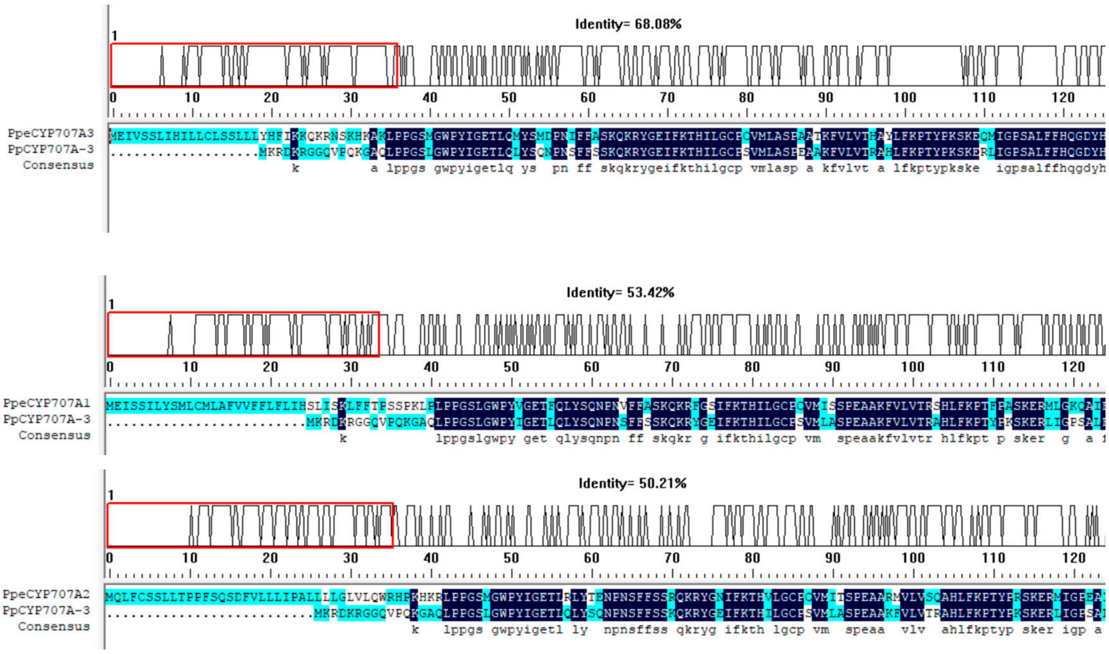

Figure S3. Multiple sequence alignment of *PpCYP707A-3* with *PpeCYP707A1* (ppa005059m), *PpeCYP707A2* (ppa005020m), *PpeCYP707A3* (ppa005226m) protein sequences use DNAMAN 6.0.

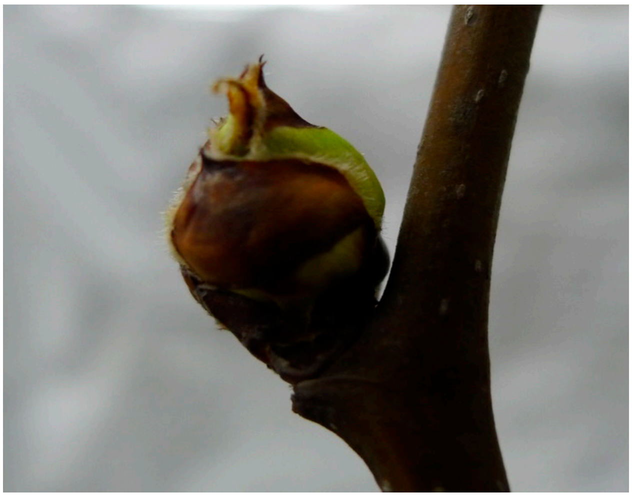

Figure S4. The photo of typical flower bud break. The beginning of bud break was defined as green leaf tips enclosing visible flowers.

Table S1. Primers used to quantify the expression levels of ABA metabolic signaling genes and reference genes.

Gene Name	Primer Sequence (5'-3')	Gene Name	Primer Sequence (5'-3')
Pbr015257.1	F: CTGTGGTTGTGGTGGACTTG	*Pbr009089.1*	F: GATCGTGGTTATCGGCTCGT
	R: GCCGACGAGAAAATGAACGA		R: TTCTCCCTAGCTTGTCCCGA
Pbr009544.1	F: ACGGTGTTGGCTTAGGGTTA	*Pbr039596.1*	F: AAGGAAGCCACTCGAGCAAA
	R: TTGCTGCTGATGTTGCTGTT		R: TTTGGACACGTGGGTCTGAG
Pbr041497.1	F: GATCCTGCTGCTCAATCTGC	*Pbr006012.1*	F: CATGGACCCACCGGATTCAA
	R: TGTCATCCAACCCAGGTGAA		R: AGCAATACCCGAACACCTGG
Pbr028792.1	F: GGCTGTTCATGTGTGCTGTA	*Pbr003186.1*	F: TGAAATACATCGAACGCGGC
	R: AGAATGGTGGCGAAACAAGG		R: TCCTCTCAAAGAGCTCTCCAC
Pbr025010.1	F: CTTTCAAACCGTGCCCTTCA	*Pbr040625.1*	F: GGCGAATCCTTACTGTACGCT
	R: GTGCGGTTCGGATCAAACAA		R: TCTATGCTCTGGGCTGGAGT
Pbr019599.1	F: CAATACACGCTGGGTCATCG	*Pbr042784.1*	F: CCAGAGTGCCGCCATCTAAT
	R: CAACAGATGCACCACCTTCC		R: GGCTTTGCATGGGTTGATCC
Pbr022745.11	F: CGGAGTCGCACATCTGAGG	*Pbr023607.1*	F: CATGGACATGCCGATCATGC
	R: TCGAATGTGTAAAGGGGTAAAGA		R: CACCGAGGACTTCTCACCTC
Pbr013576.1	F: TCGACCTCACAAAACCCAGT	*Pbr040276.1*	F: TCCCAGTACACCATACCCGA
	R: CCCATTAATTGAAACAGCACACA		R: TGTTTAGGCTGGTCACGCTC
Pbr007589.1	F: GTGAAGGAGACGCTAAGGGT	*Pbr026536.1*	F: TGTTGCAAATCCAAGGATCACC
	R: TGAGCTTGCACAGTTTCGAC		R: ATCGGGAAACTGGAGGAGGA
Pbr026157.1	F: CGTTTTCTCGTGGGCTCAAA	*Pbr004630.1*	F: CTCTTGGCAAGGAAGAGGCT
	R: TACACGCAAAGCAAGCACAT		R: AGCTGTAGTGTCTTGGGCTG
Pbr019878.1	F: TTTTCACCCGGGCTTCATTG	*Pbr019636.1*	F: TCAGCTCCCTCCAGGTTCAT
	R: GCAAATACCTCGCTGAGCAA		R: GGGATACGTGGGCTTGAACA
Pbr041795.1	F: CTTCCCGTGTGAACAAAGCA	*Pbr029414.1*	F: GATGCCCATGACTTGTAGGGTT
	R: TGTACATCAGCGCTTGGGTA		R: CGCTGCCAAATGGCATGTAT
Pbr013336.1	F: TGGGCTATTCATGTGTGCTG	*Pbr006776.1*	F: ATCTTCCAGGCACGCTGTTT
	R: AGAATGGTGGCGAATCAACG		R: GATGACCCCAATGACGTTGTC
Pbr015521.1	F: AGGTCAATCTCCACGCTTGA	*Pbr003860.1*	F: CTTCCCTCAGCCCGAAAAGT
	R: CCCTTGCCTCCTGAATTCCT		R: AAGCAGTGGTGAGGTGATGG
Pbr028942.1	F: GTCAGACCAAGAAAGACGCC	*Pbr010794.1*	F: ACTACATTCGGAGGCACCAC
	R: CGACACAAACTCACACTGCA		R: ATCTCAAGGTTCCCCTGCAC
Pbr009703.1	F: CTGTTCCACCATTGTCCCCT	*Pbr042468.1*	F: GACTCAGGACGAGTTCGACG
	R: TGCATGAATATAGGGGCGGA		R: CACGTTACAGCTCCGGATGA
Pbr017778.1	F: GCAGAACTGAACCAATTGCG	*Pbr036422.1*	F: TAACTGCAAAGGCGAGGAGG
	R: GGGTAGGGCTTCAGTTCTGT		R: CCATAGTAGCCACCCCAAGC
Pbr013616.1	F: ATAGGAATGGAGGCGCAGGA	*Pbr016128.1*	F: AGCCCTGTAAGCAAAACTCA
	R: ACACCTGCTGACAAATGGCT		R: GCATGCTGCAGCTCTTAACG
Pbr000497.1	F: CAGTTCGACAATCCGAAGGC	*Pbr019827.1*	F: GTTTTCCGAGCTGGAGGGTC
	R: GCATGACGTGCATGTTGTCG		R: AAGTTCCGGACCAACGGC
Pbr027457.1	F: GGCGGAGTACATAAGGAGGC	*PpACTIN*	F: CCATCCAGGCTGTTCTCTC
	R: TAAGGCTCCCAATGCCAAGG		R: GCAAGGTCCAGACGAAGG
